# Retrospect of the Medical Sciences

**Published:** 1843-09-30

**Authors:** 


### GALLIC ACID IN MENORRHAGIA.

Professor Simpson stated, that for the last year he had employed gallic acid in some cases of menorrhagia with the most successful results. Like all the other remedies directed against that disease, it had also occasionally failed in his hands. Some of the cases which had completely yielded under its use were of an old standing and aggravated description. He gave it during the intervals, as well as during the discharge, in doses of from ten to twenty grains per day made into pills. It had this advantage over most other anti-hemorrhagic medicines, that it had no constipating effect upon the bowels. He was first induced to use it from finding a case of very obstinate menorrhagia get well under the use of Ruspini's styptic, after many other remedies had utterly failed, and from it being alleged that gallic acid was the active ingredient in that styptic. He suggested whether the anti-hæmorrhagic properties of some of our astringent drugs may not depend upon the gallic acid as much, or more than upon the tannin which they contain, or upon the tannin becoming changed into gallic acid within the body.—*Edinburgh Monthly Journal.*

[In the Ed. Med. and Surg. Journal for July, Dr. Stevenson reports several cases of uterine hemorrhage successfully treated by gallic acid.]

### RETROSPECT OF THE MEDICAL SCIENCES.

### MORBID STATE OF THE FUNCTION OF THE SKIN IN INSANITY.

The function of the skin is frequently badly performed in insanity. The peculiar smell which some madmen have I have often heard spoken of by those who have had much experience in these cases, and I always attributed it to the uncleanliness of the patient, but since my attention has been more particularly drawn to it, I think that the peculiar odour is due to the disease; when it exists, however, it is a very unfavourable sign; the only thing I can compare it to is the smell of a person just prior to dissolution. Baths of all descriptions have very properly been recommended to ensure the due performance of this important function. In the hospital we employ hot and cold baths, the hip bath, the shower bath, pediluvia, and the douche. The tepid bath is of great service in subduing irritability and excitement. It is sometimes necessary that the patient should remain in for an hour and a half, or two hours. Ice or cold lotions to the head should be applied at the same time; it may be necessary to repeat it every day, sometimes twice daily, till some effect is produced.—*Dr. Sutherland, in Prov. Med. Journ.*

### UNHEALTHINESS OF LIVERPOOL, AND ITS CAUSES.

We take this opportunity of expressing our satisfaction at the talent and unflinching honesty with which Dr. Duncan, in his pamphlet on the "Physical Causes of the High Rate of Mortality in Liverpool," careless of bad or good report, has exposed the hygienic evils suffered by the townspeople of Liverpool, who are greatly his debtors on that account, and who will by-and-by acknowledge the fact.

The comparative tables of mortality in towns which we gave last week, quite confirm what Dr. Duncan has advanced, namely, "that, judging from the annual proportion of deaths to the population, Liverpool is the most unhealthy town in England." Indeed, it cannot well be otherwise,—for, first, *one fourth* of its inhabitants live in courts, mere *culs-de-sac*, with a narrow opening at one end, so close that the wretched tenants can scarcely see the sky, and so filthy, from accumulated excrementitious matter, as to promote, if not generate, malignant fevers; secondly, a *tenth* part of the whole population dwell in cellars, about two-fifths of which are either damp or wet; thirdly, out of two hundred and forty-three streets in Liverpool inhabited by the working classes, only *one-fifth* (in mileage) are provided with sewers; and, fourthly, the density of its population in the district so inhabited is nearly *three times* that of the population in London. Such are, in substance, the statements of Dr. Duncan (founded on authorised returns to the town council) respecting the evils that are incidental to the habitations of the industrious classes in the first port of Great Britain. The reader may make what reflections he pleases on the conduct of a corporation that has until very lately left such glaring defects unremedied. A strict enforcement of the local Act that came into operation last November (very loosely and unsatisfactorily framed, however, in our opinion) should remove the noxious agencies, and so lengthen the average life of the working population.

The physical evils, however, that have been alleged respecting Liverpool—the vicious construction of dwellings, insufficient supply or total absence of outoffices and receptacles for refuse and excrementitious matter, the absence of drains, the deficient sewerage, and the over-crowding of the population, may be charged, in a greater or less degree, against most of the leading "industrial" towns of England and Scotland, as is abundantly proved in the distressing evidence brought before the Commons' Committee on the Health of Towns, and in the very complete Local Sanitary Reports furnished by Drs. Howard, Dehane, Baker, Neil Arnott, and Laurie, and other professional men resident in great towns, to the Poor-law commissioners. Out of a large number of such statements we take a few, at random, to illustrate our meaning. Dr. E. Knight says, respecting Stafford:—

"Those parts of the town inhabited by the lower classes are without drainage, and the houses built without any regard to situation or ventilation, constructed (with usually only two small rooms) in a manner to insure the greatest return at the least possible outlay, and without any provision made for refuse dirt and excrementitious matter, which, as the least trouble, are thrown down in front of the houses, and left to putrefy and diffuse a constant malaria."—*Local Sanitary Reports*, p. 225, *from London Lancet*, Aug. 12, 1843.

### ANTAGONISM OF PHTHISIS AND INTERMITTENT FEVER.

At the sitting of the Royal Academy of Medicine, Paris, May 23d, communications were read from MM. Casimir Broussais, Bonnefonte, Michel Levy, &c. relative to the comparative non-prevalence of tubercular consumption in Algeria. According to M. Broussais the mortality from phthisis among the civil population of that country is only 1 death in 20, while in Paris it is 1 in 5; the deaths from phthisis among the European inhabitants of Algiers being 1 in 15; those among the Jews 1 in 56; and among the Arabs and Turks 1 in 20, as far as could be ascertained. In combatting the objection that individuals dying there from fever and dysentery might, had they lived, been subsequently carried off by phthisis, M. Broussais adds, that "tubercles, even in a crude state, are never found, in Algiers, in patients dying of any other disease than consumption." These remarks are in accordance with what was stated in a previous report to the Academy by M. Boudin, who says, however, that the comparative indemnity from tubercular phthisis in Algeria prevails only on the sea-coast, and where this is of a marshy nature. Where intermittent fever is frequent, phthisis does not exist. Among the due prophylactic measures against the latter disease is, therefore, the sending patients liable thereto into localities where intermittents prevail. (Assuredly, if individuals die of ague they are effectually preserved against tubercular consumption.) The isles of Hyeres and the neighborhoods of Pisa and Rome, in which marshes and low grounds are plentiful, are said to be as eligible for consumptive patients as they are liable to bring on intermittent fevers in other persons. Similar remarks apply to the Ionian islands, the shores of the Morea, the vicinity of Cadiz, the province of South Holland, the lower parts of Lancashire, and some other English counties, &c. The observations of MM. Bonnefonte and Levy tend to oppose the doctrine of antagonism between intermittents and phthisis. The former says that the conclusions of M.

Boudin are not borne out by statistical research, for that at Rome the number of persons dying of phthisis is in a ratio nearly equal to that prevalent at Paris. There the proportion is 1 to 3.41 of the deaths, while at Rome it is 1 to 3.44. M. Bonnefonte, however, admits that, to whatever cause it may be owing, the climate of Algiers seems to be highly suitable to the consumptive; for the mortality there from phthisis, among the military force, is only 1 to 19.55 of the total deaths, and 1 to 17.1 in the civil population. Constantine, Medeah, and the other towns in the same regency, appear to share in this indemnity. M. Levy asserts that Strasbourg, surrounded by marshes, is subject to the prevalence of both consumption and intermittent fever, and that he has met with various instances of phthisis in the marshy lands of both Corsica and Greece. But with respect to the prevalence of the two maladies at Strasbourg, it was advanced by M. Boudin, at the next sitting of the Academy, that, while acknowledging the prevalence of both at that place, the localities of their origin appeared to be different, one being endemic only in the marshy country without the walls, and the other in the citadel alone.—*Lon. Lancet, from Bull. de l'Acad. Royale.*

EXHALATION OF CARBONIC ACID FROM THE BODY.

Professor Scharling, of Copenhagen, recently made some experiments to ascertain the quantity of carbonic acid exhaled from the human frame by the lungs and surface of the skin in twenty-four hours. The method he employed was the following:

The subjects of experiment were confined in an air-tight box, wherein they were perfectly at ease, being enabled to speak, eat, sleep, or read, without inconvenience; a constant current of atmospheric air was admitted into the box, and the deteriorated gases abstracted by means of an air-pump. The air withdrawn was conducted into a proper arrangement of bottles, some containing sulphuric acid, others a solution of caustic potash. The quantity of carbonic acid, both previously and subsequent to each operation, was carefully ascertained by being received into three graduated tubes. The results were as follows:

The professor himself, aged thirty-five years, exhaled 219 grammes, or 7.7 ounces avoird. during twenty-four hours, seven of which were spent in sleep; a soldier, twenty-eight years of age, exhaled 339.728 grammes—8.45 ounces; a lad of sixteen, 234.379 grammes—7.9 ounces; a young woman, aged nineteen, 165.347 grammes—5.83 ounces; a boy, nine and a half years old, 133.126 grammes—3.069 ounces; a girl, ten years old, 125.42 grammes—4.42 ounces. In the two last, the period allotted to sleep was nine hours.

From these experiments Professor Scharling deduces, that males exhale more carbonic acid than females, and children comparatively more than adults. He also finds that less of the gas is given off during the night than during the day, and that in certain cases of disease, which he does not specify, less carbonic acid is formed then during the healthy state.—*Ibid, from Phil. Mag.*

POPULATION OF BELGIUM.

The census of this (relatively) most populous country in Europe for 1842, is, we believe not yet published. At the end of 1841 its eighty-six towns contained a population of 1,006,117, and its rural districts 3,111,485 persons; total, 4,117,602. Births in 1841, 37,222 in the towns, and 106,438 in the other communes. Deaths, 30,391 in the former, and 72,227 in the latter. Marriages, 28,963. The number of females, as usual, surpassed that of the males; and the number of widows was double that of widowers. As many males were below as above 23, and as many females below as above 25 years of age. The number of males between 20 and 50 amounted to 778,381.—*Ibid, from Echo du Monde Savant.*

M. PELIGOT ON THE CHEMICAL COMPOSITION OF TEA.

At the sitting ef the Academy of Science, July 17th, M. Peligot read a paper on the chemical composition of tea. As this substance (says he) contains more nitrogenised matters than any other substance used as food (?), it may be considered an article of diet of high importance. On instituting a comparison between ordinary sugared tea and broth of moderate richness, M. Peligot has arrived at the following conclusions:—The ordinary broth of the Dutch East India Gompany's service, as analysed by the French academicians, has been found to contain, in a quart, 15 grammes (nearly half an ounce) of soluble organic matters, and 9 grammes (2¼ drachms) of soluble inorganic matter (salt, &c.). The presumed quantity of nitrogen in the organic matters was not determined by the academicians, but M. Peligot estimates it at about eight per cent., which would give about eighteen grains by weight in a litre or quart of broth. Tea made with five drachms to the quart of water and sugared, gave the following results:—Dry residue of infusion of tea, 1⅗th drachms; sugar, 6½ drachms. These products comprise about fifteen grains of *theine*, and four and a half grains of nitrogen. Thus, if broth be the richer in nitrogen, tea-infusion (with sugar!) contains solid matter in greater quantity; and tea may, therefore, be not only an excitant, but an article of considerable nutrient quality.—*Lancet.*

Medical practitioners are cautioned against *a spurious preparation of Mercury*, presented in the form of a yellow powder, which is at present being sold for mixing with ointment, to produce an imitation of the *ung. hydr. nitr.* of the Pharmacopœia. The preparation (as the *Chemist* argues) bears the mark of fraud in its very name. It is called *subnitrate* of mercury; whereas, in the ointment, as made according to the direction of the Pharmacopœia, "It is quite evident that a *super*-salt, with a large excess of acid, and not a subnitrate is produced. The peculiar changes which the metallic solution and the fatty matters undergo, are but imperfectly understood, but it has been generally supposed that intimate combination takes place—that a nitrate of the peroxide of mercury remains in close connection with the oil, &c., while the latter are partly oxidated, and elaïdate of the oxide of glycerule, and other new compounds result, and in the newly made ointment a little free acid is also present. The spurious preparation in question possesses the quantity, neither of mercury nor of nitric acid, employed in the preparation of the *ung. hyd. nitr.*, and is wanting in many of its most valuable properties.—*Ibid.*

A German practitioner strongly recommends spirit of ammonia as a local application to bruises, contusions, &c., as a valuable resolutive agent, capable of fulfilling all the objects for which ordinary refrigerants are used.—*Ibid.*

